# Bridging Innovation and Practice in Type 2 Diabetes Mellitus: Novel Antidiabetic Therapies and the Expanding Role of Community Pharmacists

**DOI:** 10.3390/ph19020271

**Published:** 2026-02-05

**Authors:** Marios Spanakis, Agapi Fournaraki, Frantzeska Nimee, Christos Kontogiorgis, Emmanouil K. Symvoulakis

**Affiliations:** 1 Department of Social Medicine, School of Medicine, University of Crete, 70013 Heraklion, Greece; esymvoulakis@uoc.gr; 2 Community Pharmacists Association of Heraklion, 71201 Heraklion, Greece; 3 Community Pharmacists Association of Rethymno, 74132 Rethymno, Greece; pharmagapi@gmail.com; 4 Laboratory of Hygiene and Environmental Protection, Department of Medicine, Democritus University of Thrace, 68100 Alexandroupolis, Greece; fnimee@hua.gr (F.N.); ckontogi@med.duth.gr (C.K.); 5 Community Pharmacists Association of Athens, 10436 Athens, Greece

**Keywords:** type 2 diabetes mellitus, antidiabetic agents, GLP-1, SGLT2 inhibitors, DPP-4 inhibitors, primary care, community pharmacy, pharmacovigilance, real-world evidence, pharmacist-led interventions

## Abstract

Diabetes mellitus, particularly type 2 diabetes mellitus (T2DM), represents a rapidly expanding global health challenge with substantial public health and economic consequences. Recent advances in antidiabetic therapy—including dipeptidyl peptidase-4 (DPP-4) inhibitors, glucagon-like peptide-1 receptor agonists (GLP-1 RAs), dual GIP/GLP-1 receptor agonists, and sodium–glucose cotransporter-2 (SGLT-2) inhibitors—have transformed diabetes management by providing benefits beyond glycemic control, such as cardiovascular and renal protection, weight reduction, and improved quality of life. As the therapeutic landscape becomes increasingly complex and patient-centered, ensuring the safe and effective use of these agents in real-world settings has emerged as a key concern for pharmacoepidemiology and pharmacovigilance. Community pharmacists, as highly accessible healthcare professionals, play an expanding role in diabetes care through medication optimization, patient education, adherence support, and monitoring of adverse drug reactions in primary care. Evidence from systematic reviews and meta-analyses indicates that pharmacist-led interventions improve glycemic outcomes, enhance self-care behaviors, and facilitate the appropriate adoption of contemporary antidiabetic therapies. This narrative review synthesizes current evidence on novel pharmacological treatments for T2DM and examines the evolving contribution of community pharmacists in translating therapeutic innovation into routine practice. Barriers to implementation and future perspectives for integrating pharmacist-led services into diabetes management and pharmacovigilance frameworks are also discussed.

## 1. Introduction

Diabetes mellitus (DM) represents one of the most rapidly escalating global health crises of the twenty-first century. Currently, it affects approximately 537 million people worldwide, and this number is projected to reach 783 million by 2045. Among all cases of diabetes, more than 90% are type 2 diabetes mellitus (T2DM) [1,2]. The widespread and increasing prevalence of diabetes on a global scale, approaching pandemic proportions, makes it a major public health issue with significant economic, social, and healthcare consequences that go far beyond mere statistical figures [3,4,5].

T2DM is a chronic, multifactorial metabolic disorder arising from the interplay of genetic predisposition and environmental factors. It is characterized by insulin resistance combined with progressive β-cell dysfunction in pancreas, resulting in persistent hyperglycemia. Chronic hyperglycemia leads to microvascular complications, including retinopathy, nephropathy, and neuropathy, as well as macrovascular disease involving coronary, cerebral, and peripheral arteries. Consequently, T2DM is strongly associated with increased risk of cardiovascular disease (CVD) and chronic kidney disease (CKD), which remain the leading causes of morbidity and mortality among affected individuals [1,4,5,6,7,8]. The close interrelationship between T2DM, obesity, CVD, and CKD have led to the recognition of the cardiorenal–metabolic (CKM) syndrome, a unified pathophysiological construct associated with synergistic disease progression and adverse clinical outcomes [9,10,11,12].

From a pharmacoepidemiological perspective, T2DM represents a chronic condition characterized by lifelong pharmacological exposure, high disease prevalence, and extensive population-level drug utilization. Effective diabetes management requires an appreciation of this complexity and extends beyond the control of biochemical markers alone. Prevention of disease progression and complications relies on individualized, long-term strategies combining lifestyle modification, pharmacological therapy, and sustained self-management within interdisciplinary models of care [4,6,7,13,14]. In real-world settings, however, the chronic nature of T2DM, frequent comorbidities, and polypharmacy substantially increase the risk of medication-related problems, including adverse drug reactions (ADRs), drug–drug interactions (DDIs), and suboptimal adherence—key concerns for pharmacovigilance systems [15,16].

Historically, pharmacological management relied on conventional glucose-lowering agents such as metformin, sulfonylureas, thiazolidinediones, and insulin. While these therapies remain foundational, they present limitations in addressing the broader pathophysiology of T2DM and its cardiometabolic consequences. Achieving durable glycemic control remains challenging, particularly in patients with advanced disease and multiple comorbidities. As T2DM is increasingly recognized as a systemic disorder, contemporary therapeutic goals have expanded to include cardiovascular and renal risk mitigation, necessitating a re-evaluation of traditional, glucose-centric, treatment paradigms [17,18,19]. In this context, novel antidiabetic therapies—including dipeptidyl peptidase-4 (DPP-4) inhibitors, glucagon-like peptide-1 (GLP-1) receptor agonists, dual glucose-dependent insulinotropic polypeptide (GIP)/GLP-1 receptor agonists, and sodium–glucose cotransporter-2 (SGLT2) inhibitors—have reshaped, and will continue to do so, diabetes care [20]. These agents provide multifaceted benefits encompassing glycemic control, weight reduction, and cardiovascular and renal protection, supported by large trials and rapidly translated into clinical practice [1,2,6,21]. Their accelerated adoption leads in the necessity for post-marketing surveillance to evaluate long-term safety, real-world effectiveness, and rare or delayed adverse outcomes across diverse populations.

Despite these pharmacological advances, optimal diabetes management continues to depend on coordinated primary healthcare systems and effective interdisciplinary collaboration [22,23]. Contemporary approaches increasingly integrate novel pharmacological agents, digital health technologies, and multidisciplinary care models aimed at reducing complications, hospitalizations, and long-term healthcare burden. Within this framework, community pharmacists (CPs) as the most accessible healthcare professionals—play an increasingly important role that extends beyond medication dispensing. Through continuous patient engagement, medication optimization, identification and reporting of adverse drug reactions (ADRs), and support for adherence and self-care, pharmacists contribute directly to real-world drug safety monitoring and pharmacoepidemiological evidence generation. As therapeutic options for T2DM expand and patients increasingly present with multiple comorbidities, polypharmacy has emerged as a central challenge in diabetes management. This complexity underscores the critical role of pharmacists in medication reconciliation, identification of drug-related problems, and optimization of individualized treatment regimens. Evidence indicates that pharmacist-led interventions improve glycemic outcomes, reduce medication-related problems, and enhance the safe implementation of complex and innovative therapeutic regimens in routine care [24,25].

The aim of this work is to synthesize current evidence on modern pharmacotherapy for T2DM, with emphasis on benefits beyond glycemic control, and to examine the evolving role of CPs as key contributors to pharmacoepidemiology and pharmacovigilance within primary healthcare systems. By situating pharmacist-led interventions within real-world drug utilization and safety frameworks, this review highlights their potential to improve clinical outcomes, patient safety, and health system performance in contemporary diabetes care.

## 2. Novel Therapies for T2DM

**Figure 1:**
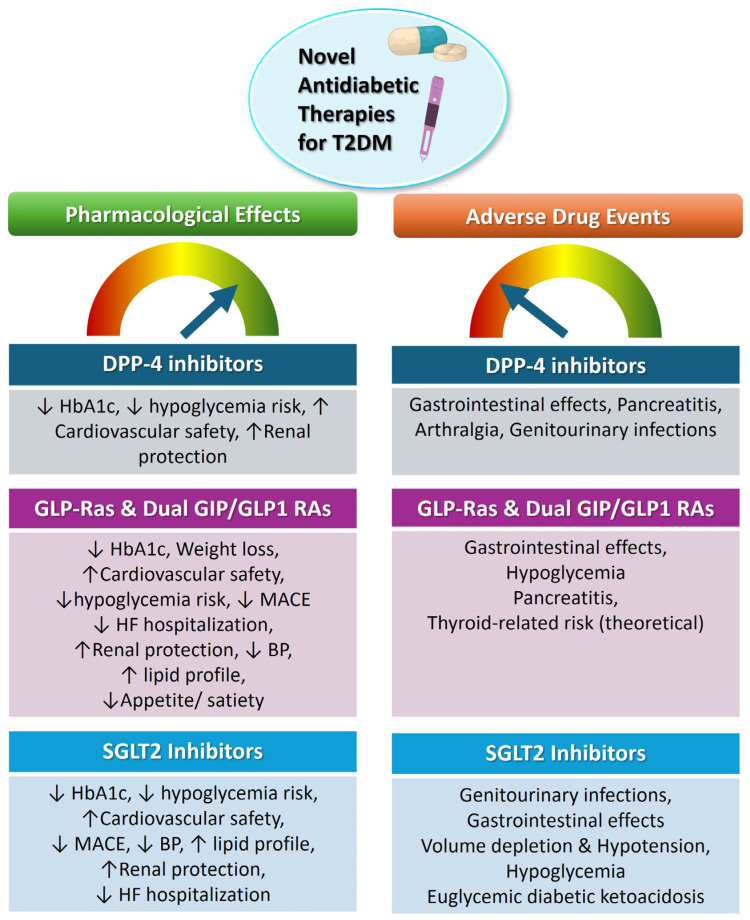
Comparative pharmacological effects and adverse events of novel antidiabetic drug classes (↑: increase; ↓: decrease, ↑ lipid profile: improved lipid profile).

The development of novel glucose-lowering agents has shifted therapeutic goals toward comprehensive cardiorenal–metabolic protection (Table 1). As their use expands across diverse populations and healthcare settings, real-world effectiveness, safety, and utilization patterns have become key concerns for pharmacoepidemiology and pharmacovigilance. Current therapeutic positioning is following international consensus recommendations, including guidelines from American Diabetes Association (ADA), European Association for the study of Diabetes (EASD) and European Society of Cardiology (ESC) [26]. These frameworks emphasize patient-centered treatment selection based on comorbidities such as established atherosclerotic cardiovascular disease (ASCVD), heart failure (HF), CKD, and obesity, rather than a glucose-centric escalation model. While metformin remains first-line therapy for most individuals without compelling comorbidities, early initiation of SGLT2 inhibitors or GLP-1 receptor agonists is recommended in patients with established or high risk of ASCVD, HF, or CKD, irrespective of baseline HbA1c. Accordingly, DPP-4 inhibitors retain a role in selected populations where tolerability and safety are paramount. Dual incretin agonists are emerging as high-efficacy options, with ongoing studies expected to further refine sequencing strategies. Nevertheless, treatment positioning should explicitly address contraindications and monitoring requirements, including renal function thresholds and dose adjustments, gastrointestinal event considerations with GLP-1 receptor agonists, HF related signals with specific DPP-4 inhibitors, and the risk of euglycemic diabetic ketoacidosis with SGLT2 inhibitors, alongside appropriate patient education and follow-up. This structured, guideline-concordant approach ensures clinical coherence, safety, and relevance to contemporary T2DM care. Figure 1 presents the main pharmacological outcomes and the adverse drug events associated with each drug class.

### 2.1. Dipeptidyl Peptidase-4 (DPP-4) Inhibitors

DPP-4 inhibitors, including sitagliptin, linagliptin, vildagliptin, saxagliptin, and alogliptin, are widely used oral agents for T2DM. Their mechanism involves inhibition of the DPP-4 enzyme, thereby increasing endogenous incretin hormones (GLP-1 and GIP), which enhances glucose-dependent insulin secretion and suppresses glucagon release, resulting in lower blood glucose levels [1,27].

DPP-4 inhibitors can be administered independently of meals and are available in fixed-dose combinations with metformin. They are well-tolerated, with minimal DDIs, and are suitable for patients with renal impairment and polypharmacy.

#### 2.1.1. Cardiovascular and Renal Protection of DPP-4 Inhibitors 

Large clinical trials demonstrate that DPP-4 inhibitors effectively reduce HbA1c, while maintaining a neutral cardiovascular safety profile. The TECOS trial involving over 14,000 patients showed that sitagliptin improved glycemic control (achieving lower HbA1c) without increasing cardiovascular risk [28]. Similarly, the SAVOR-TIMI 53 that enrolled more than 16,000 patients demonstrated cardiovascular safety for saxagliptin [29]. Furthermore, CARMELINA and CAROLINA trials demonstrated the cardiovascular safety and efficacy of linagliptin in reducing HbA1c levels [30,31]. Although modest reductions in blood pressure and lipid parameters have been reported, these effects appear heterogenous across studies and are generally considered secondary rather than clinically decisive outcomes. Importantly, while cardiovascular safety has been consistently established, DPP-4 inhibitors do not confer cardiovascular risk reduction. Saxagliptin and alogliptin are not recommended in patients with HF, whereas potential renal benefits, particularly with linagliptin, have been observed. Cost remains a limitation to broader use [1,27,32]. Overall, the glycemic efficacy and cardiovascular safety of DPP-4 inhibitors is evident especially for patients whom hypoglycemia avoidance and tolerability are priorities. As for renal protection, DPP-4 inhibitors have been associated with reductions in albuminuria, reflected by decreases in the albumin-to-creatinine ratio, suggesting a possible nephroprotective effect. However, this evidence remains largely indirect, and larger prospective clinical trials specifically designed to assess renal outcomes are required to confirm and better define their role in renal protection [33,34]. Potential cardiometabolic pleiotropic effects remain inconsistent across trials and should be interpreted cautiously, especially when compared with agents demonstrating proven cardiovascular or renal benefit.

#### 2.1.2. Adverse Events of DPP-4 Ihibitors

Adverse events are rare, with a low risk of hypoglycemia and no effect on body weight. Generally, they are well-tolerated with a low incidence of gastrointestinal effects. However, they may increase the risk of upper respiratory and urinary tract infections, and rarely acute pancreatitis or severe arthralgia [21,35,36,37].

### 2.2. Glucagon-like Peptide-1 (GLP-1) Receptor Agonists and Analogs

GLP-1 is an incretin hormone secreted by L-cells in the ileum and colon in response to nutrient intake, primarily glucose and lipids. It exerts its effects via GLP-1 receptors present in multiple organs, promoting glucose-dependent insulin secretion, suppressing glucagon release, delaying gastric emptying, reducing appetite, and improving insulin sensitivity. Native GLP-1 is rapidly degraded by dipeptidyl peptidase-4 (DPP-4), resulting in a short half-life. Consequently, GLP-1 receptor agonists (GLP-1 RAs) have been developed to resist DPP-4 degradation and prolong activity [38,39,40,41,42,43]. The development of GLP-1 analogs signifies a milestone in diabetes therapy shifting the standard of care for patients with established cardiovascular disease or obesity. Their ability to address multiple interconnected cardiometabolic conditions with a single therapy has made them a cornerstone of modern chronic disease management [44,45].

#### 2.2.1. Classification and Indications

GLP-1 RAs are now central to T2DM management. These agents stimulate insulin secretion in a glucose-dependent manner, minimizing hypoglycemia risk, and are administered subcutaneously once daily or weekly [17,32,39,46,47,48].

There are two main subcategories:

- GLP-1 analogs: Structurally similar to endogenous GLP-1 and resistant to DPP-4, with prolonged activity. Examples include liraglutide (once daily), dulaglutide, and semaglutide (once weekly)
- GLP-1 receptor agonists: Structurally distinct from endogenous GLP-1 but bind to and activate GLP-1 receptors. Examples include lixisenatide (once daily) and exenatide (once weekly).

#### 2.2.2. Cardiovascular and Renal Protection of GLP-1 RAs

GLP-1 RAs reduce major adverse cardiovascular events and are recommended for patients with T2DM and established cardiovascular disease or multiple cardiovascular risk factors (age, hypertension, hyperlipidemia, smoking, obesity, etc.). Studies (LEADER, REWIND, SUSTAIN-6) have demonstrated cardiovascular benefit beyond their safety, making them first-line therapy in high-risk individuals [1,12,39,49]. As for renal protection, GLP-1 RAs slow progression of diabetic kidney disease and reduce albuminuria, potentially via antioxidative, anti-inflammatory, and antifibrotic effects [50,51,52,53]. Apart from cardiorenal protection, GLP-1 RAs have been associated with improvement in hepatic steatosis, lipid metabolism, and blood pressure regulation, as well as potential neuroprotective effects reported mainly in preclinical studies and observational human data [45,54]. Overall, the cardiovascular benefit of several GLP-1 receptor agonists in patients with established ASCVD is well established. In contrast, neurocognitive and mental health effects remain exploratory, warranting further investigation in adequately powered RCTs.

#### 2.2.3. Weight Loss with GLP-1 RAs

GLP-1 RAs are a milestone for treatment of obesity. They have fundamentally shifted weight management from a behavioral-discipline model to a holistic approach for a chronic-disease medical issue. Liraglutide and Semaglutide promote strong weight loss and have been approved as anti-obesity medications for individuals both with and without diabetes. The Semaglutide Treatment Effect in People with Obesity (STEP) trials for semaglutide demonstrated unprecedented efficacy for weight loss [55,56,57]. GLP-1 RAs induce weight loss through appetite suppression acting on the Central Nervous System (CNS) to suppress appetite and increase satiety alongside slowing down gastric emptying, leading to reduced food intake and prolonged fullness, which collectively promote sustained weight loss. Till today clinical trials indicate varying degrees of weight reduction, with semaglutide and liraglutide achieving substantial effects, including in non-diabetic individuals [58,59,60].

#### 2.2.4. Adverse Effects of GLP-1 RAs

The most common adverse effects are gastrointestinal (nausea, vomiting, diarrhea, flatulence), which are typically transient. Rarely, GLP-1 RAs are associated with pancreatitis and medullary thyroid carcinoma. Exenatide and lixisenatide are contraindicated in advanced renal impairment and severe gastrointestinal disease; liraglutide and dulaglutide are contraindicated in patients with a history of medullary thyroid carcinoma or multiple endocrine neoplasia. The risk of hypoglycemia is low unless combined with other hypoglycemic agents [61,62,63,64,65].

### 2.3. Dual GIP and GLP-1 Receptor Agonist (Tirzepatide)

Tirzepatide is a modified peptide that binds both GLP-1 and GIP receptors. Administered once weekly, titration begins at 2.5 mg, with maintenance doses up to 15 mg [66]. Clinical trials (SURPASS and meta-analyses of seven RCTs) demonstrate dose-dependent superiority in glycemic control and HbA1c reduction compared to placebo, long-acting GLP-1 RAs (semaglutide), and basal insulin (glargine) [40,42,67,68,69]. Within the modern T2DM treatment continuum, dual incretin agonists represent a highly effective therapeutic option; nevertheless, the progressive nature of the disease, characterized by gradual β-cell dysfunction, means that insulin therapy remains an important complementary component in long-term management [70].

#### 2.3.1. Cardiovascular and Renal Protection of Dual GIP and GLP-1 RAs

Tirzepatide demonstrates greater renoprotection compared to basal insulin, with reductions in blood pressure (5–6 mmHg), triglycerides, LDL, VLDL cholesterol, and increases in HDL. In SURMOUNT-1, tirzepatide also reduced predicted risk of ASCVD in obese or overweight non-diabetic subjects [71,72].

#### 2.3.2. Weight Loss with Dual GIP and GLP-1 RAs

Similar to GLP-RAs, tirzetapide represents a milestone for weight management and the treatment of obesity. The SURPASS and SURMOUNT-1 trials have demonstrated significant, sustained, dose-dependent weight loss with tirzepatide, including in non-diabetic individuals. Reductions of 15% to 20% of total body weight have been reported, leading to comparisons with bariatric surgery outcomes [73]. However, these comparisons are indirect, population- and endpoint-specific, and based on limited follow-up durations, and therefore do not imply equivalence across long-term metabolic or clinical outcomes. Real-world data suggest more modest weight reductions, closer to 5–7% over two years, compared with bariatric surgery, which typically achieves 25–30% total body weight loss within the first year with greater long-term durability [74,75]. Meta-analyses show all doses seem to be superior to comparators (basal insulin, GLP-1 RAs), with weight loss ranging from 5.4 to 11.7 kg for doses of 5–15 mg [40,67,69,71,72,76,77]. In summary, dual incretin agonists represent a significant therapeutic advance for glycemic control and weight management. Long-term cardiovascular, renal, and durability outcomes are actively being investigated and will further define their positioning relative to established therapies [78].

#### 2.3.3. Adverse Effects of Dual GIP and GLP-1 RAs

The most common adverse effects are gastrointestinal, primarily nausea, vomiting, and diarrhea, occurring in a dose-dependent manner [66,67].

### 2.4. Sodium-Glucose Cotransporter-2 (SGLT2) Inhibitors

SGLT2 inhibitors competitively block renal SGLT2 proteins, reducing glucose and sodium reabsorption, and increasing urinary excretion. This insulin-independent mechanism lowers plasma glucose with a low risk of hypoglycemia [79].

Dapagliflozin, canagliflozin, and empagliflozin are key agents. SGLT2 inhibition is associated with a state of relative glucose “deprivation” or a negative energy balance at the cellular level. This leads to glycosuria, improved glycemic control, natriuresis, reduced albuminuria, blood pressure improvement, and weight loss via enhanced lipid metabolism. Ketone production is increased, providing favorable energy substrates for renal and myocardial cells [79,80].

#### 2.4.1. Cardiovascular and Renal Protection of SGLT2 Inhibitors

SGLT2 inhibitors offer significant cardiovascular and renal advantages, reducing mortality, HF hospitalizations, major adverse cardiovascular events (MACE), kidney disease progression, the incidence of end-stage renal disease and mortality for chronic kidney disease in patients with or without diabetes [52,80,81,82]. Large clinical trials have shown consistent benefits. although effects on MACE vary across studies and appear influenced by baseline cardiovascular risk EMPA-REG OUTCOME (empagliflozin) demonstrated a 14% reduction in MACE, with notable decreases in CV death and HF hospitalizations; CANVAS/CANVAS-R (canagliflozin) showed similar reductions in MACE and HF risk; DECLARE-TIMI 58 (dapagliflozin) reported a 17% drop in cardiovascular-related death or HF hospitalization; VERTIS CV (ertugliflozin) confirmed HF benefit despite neutral effects on MACE [83,84,85,86]. Renal protection has been further validated in dedicated studies: CREDENCE and DAPA-CKD showed major decreases in kidney disease progression, and EMPA-KIDNEY found broad renal benefit across chronic kidney disease populations, confirming these effects as class-wide [87,88,89]. In summary, evidence for HF and renal protection with SGLT2 inhibitors is strong and consistent. In contrast, heterogeneity in ASCVD outcomes underscores the importance of patient stratification when selecting therapy.

#### 2.4.2. Weight Loss with SGLT2 Inhibitors

Weight loss of 2–4 kg is typical after 6–12 months of therapy. Additional benefits include improved erythropoiesis, anemia correction, hepatic function, and reduced adiposity and uric acid levels [6,12,17,32,52,79].

#### 2.4.3. Adverse Effects of SGLT2 Inhibitors

Common adverse effects are genitourinary infections, particularly genital mycotic infections in women, (due to increased glucose in the urine, or glycosuria), dehydration (also called volume depletion) due to natriuresis and concomitant water loss (especially with concurrent diuretic use), and orthostatic hypotension due to osmotic diuresis. Euglycemic diabetic ketoacidosis has been reported in some cases [79,90].

## 3. Evidence on Pharmacists-Led Interventions on T2DM

Pharmacists represent the third largest healthcare profession globally and are among the most accessible medication experts within healthcare systems [32]. Their role has progressively expanded beyond traditional dispensing and counseling to encompass preventive services, chronic disease management, and public health interventions. Frequent patient contact—often without the need for appointments—positions CPs as key facilitators of continuity of care and early intervention, particularly within primary healthcare settings [24,91]. Furthermore, there is growing body of evidence suggesting that collaboration with primary care physicians leads in improved outcomes for patients with diabetes [92,93,94,95,96].

The rapid advancement of pharmacotherapy for T2DM, particularly with the introduction of SGLT2 inhibitors and GLP-1 receptor agonists, has significantly altered clinical practice. These developments create new opportunities and challenges for medication optimization in primary care. Within this context, CPs play a pivotal role in translating pharmacological innovation into effective real-world practice. Through structured, pharmacist-led interventions, innovative therapies can be safely and effectively integrated into routine care, supporting individualized, patient-centered treatment strategies [24]. A growing body of evidence demonstrates that pharmacist-led interventions improve both preventive and therapeutic outcomes in diabetes management. These interventions contribute to improved glycemic control, enhanced adherence, reduced medication-related problems, and better patient self-management [24,91,97]. The evidence is summarized in a PICO-based table of systematic reviews and meta-analyses (Table 2).

### Pharmacoepidemiological Data on Community Pharmacists-Led Interventions in T2DM

A recent systematic review and meta-analysis by Coutureau et al. demonstrated that pharmacist-led interventions in primary care significantly reduced HbA1c levels compared with usual care. Core components of these interventions included patient education and structured medication regimen reviews, underscoring the value of pharmacist involvement in improving real-world glycemic outcomes [98]. Earlier work by Deters et al. similarly reported significant HbA1c reductions following community pharmacist–led pharmaceutical care interventions in patients with both T1DM and T2DM. These interventions were patient-centered and interdisciplinary, incorporating medication review, individualized goal setting, and structured feedback to physicians [99].

Presley et al., in a large-scale systematic review and meta-analysis encompassing 59 studies across six continents, confirmed that pharmacist-led educational and behavioral interventions consistently improved medication adherence and achievement of glycemic targets among adults with diabetes. Education emerged as a central and recurring component of effective intervention strategies [100]. Further evidence from a systematic review by Al Assaf et al. demonstrated that pharmacist-led interventions in community settings significantly enhanced adherence to antidiabetic therapy and improved HbA1c levels in approximately 90% of included studies. Interventions ranged from face-to-face counseling and structured educational sessions to remote telephone-based follow-up [91]. Similarly, Bukhsh et al. reported that pharmacist-led educational interventions significantly improved self-care behaviors, including blood glucose monitoring, dietary habits, and foot care—and were associated with meaningful reductions in HbA1c among patients with T2DM [101]. Furthermore, Pousinho et al., in their systematic review of 36 randomized controlled trials (RCTs) including 5761 patients, showed that pharmacist-led interventions achieved significantly greater HbA1c reductions than usual care, with pooled mean differences of approximately −0.7% to −0.8% and reductions reaching up to ~−2.0% in individual studies. Effective interventions consistently combined individualized education, structured medication review, and regular follow-up, and were frequently associated with improvements in adherence and other cardiometabolic risk factors [102]. Similarly, van Eikenhorst et al., in a systematic review of 24 RCTs for 3610 patients, showed that pharmacist-led self-management interventions achieved a pooled mean HbA1c reduction of 0.71% versus usual care, alongside improvements in blood pressure, BMI, lipid parameters, medication adherence, diabetes knowledge, and self-management behaviors, highlighting the added value of pharmacist-supported care in ambulatory settings [103]. The positive impact of pharmacists-led interventions was also demonstrated in the systematic review of Blanco-Vega et al., who, in their analysis of 9 trials, showed positive effects from pharmacotherapeutic follow-up and patient education, particularly in medication management (adherence, problem-solving) leading to improved glycemic control (HbA1c) and fasting blood glucose (FBG), cardiovascular biomarkers, better self-care, and enhanced knowledge [104].

Beyond diabetes alone, pharmacists play a key role in managing metabolic syndrome, where T2DM and prediabetes represent core components. A systematic review of Al AdAwi et al. showed that pharmacist participation within multidisciplinary teams improved achievement of metabolic syndrome targets, increased reversion to non-metabolic syndrome status, and enhanced medication adherence [105].

## 4. Current Framework for Interventions of Community Pharmacists

### 4.1. Patient Identification, Treatment Initiation and Follow-Up

**Figure 2:**
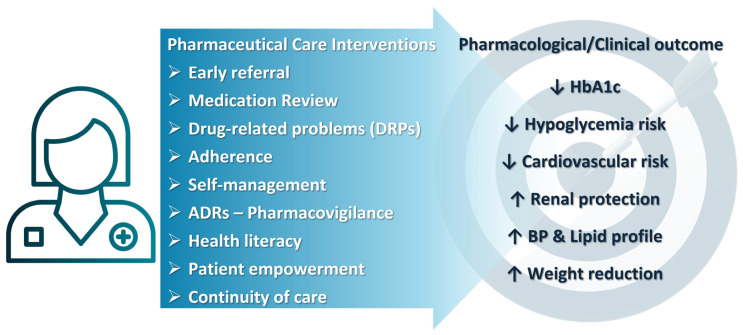
Potential CP interventions to achieve therapeutic goals in the management of T2DM. (↑: increase; ↓: decrease, ↑ BP & lipid profile: improve BP & lipid profile).

CPs contribute to early identification of individuals with prediabetes or undiagnosed diabetes and facilitate timely referral to physicians for diagnostic confirmation and therapeutic initiation [106]. They also support prevention and management of metabolic syndrome and, in collaboration with prescribers, contribute to the initiation of evidence-based therapies, including GLP-1 receptor agonists and SGLT2 inhibitors [105,107]. Their frequent patient contact and longitudinal knowledge of patient histories enable early recognition of cardiovascular and renal comorbidities and informed therapeutic recommendations [12,24] (Table 3). Figure 2 briefly presents CP interventions and their outcome as to T2DM treatment outcome.

### 4.2. Medication Review, Optimization of Antidiabetic Regimens and Deprescribing

CPs play a central role in optimizing antidiabetic regimens through structured medication review (MR) interventions (Table 3). According to the Pharmaceutical Care Network Europe (PCNE), MR is a systematic evaluation of a patient’s medicines aimed at optimizing use and improving health outcomes by identifying drug-related problems (DRPs) and proposing targeted interventions [131]. In routine community pharmacy practice, this process enables CPs to support therapeutic transitions from older agents—such as sulfonylureas or basal insulin—to newer therapies with demonstrated clinical, humanistic, and economic benefits, while also facilitating regimen simplification and deprescribing where appropriate [132]. By detecting and addressing DRPs, clinically relevant DDIs, and inappropriate polypharmacy, CP-led medication review contributes to improved adherence and reduced risk of ADRs. They also facilitate regimen simplification and deprescribing where appropriate, thereby improving adherence and improving therapy outcome [108,109,110,111,133]. Real-world evidence from the DIATHEM study demonstrated that pharmacist-led MR type 2a and type2b in older patients with T2DM significantly reduced the number of DRPs—many related to antidiabetic therapy resulting in a significant reduction in DRPs per patient and a modest but significant decrease in overall medication burden. Importantly, these outcomes were achieved under routine care conditions and through interdisciplinary collaboration with physicians, underscoring the practical value of community pharmacist–led medication review in improving medication safety and therapeutic optimization in T2DM [112,133].

### 4.3. Patient Education on Pharmacotherapy and Self-Care Activities

CPs enhance patient understanding of pharmacotherapy by providing clear, tailored information on mechanisms of action, expected benefits, administration techniques, dosing schedules, and potential adverse effects [108] (Table 3). The program Community-Based Value Initiative (CBVI) showed that pharmacist-led interventions—comprising education on diabetes, nutrition, blood glucose, cardiovascular risk, cholesterol, self-care, and medication management—resulted in a 0.5% reduction in HbA1c over six months, with complete patient satisfaction [113]. This is also related to patient empowerment for self-care activities CPs provide structured education on essential self-care behaviors, including physical activity, nutrition, glucose monitoring, medication adherence, and risk reduction strategies.

Pharmacist-led self-care education has been consistently associated with reductions in HbA1c, improved quality of life, and lower complication rates among patients with diabetes [34,35,40]. Furthermore, evidence from other pharmacist-based diabetes intervention models indicates that structured educational modules combined with medication optimization and follow-up support significantly improve patients’ knowledge of their condition and health literacy, self-care practices, medication adherence, and glycemic control over a six-month period [114]. Such educational interventions have consistently been associated with improved adherence, reduced medication errors, and fewer treatment-related complications [101,115]. The expanding availability of advanced therapies, such as GLP-1 RAs, underscores the need for equitable, integrated, and sustainable care models that combine pharmacological treatment with health promotion, disease prevention, and coordinated, community-based interventions targeting both high-risk individuals and the broader population. Considering lifestyle adoptions, CPs can also contribute to nutritional counseling by reinforcing evidence-based dietary recommendations, supporting weight management strategies, and aligning nutritional choices with prescribed pharmacotherapy. Through regular patient interactions, pharmacists can promote adherence to dietary plans, identify barriers to lifestyle modification, and provide tailored advice that complements medical nutrition therapy delivered by the broader healthcare team [134,135,136,137].

### 4.4. Safety Monitoring, Pharmacovigilance, and Interdisciplinary Management of ADRs

CPs are integral to the early detection and management of ADRs associated with modern antidiabetic therapies, including gastrointestinal effects related to GLP-1 receptor agonists and genitourinary infections, dehydration, or hypotension associated with SGLT2 inhibitor [64,65,116,117,118]. For SGLT2 inhibitors, CPs are well positioned to recognize early signs of GI related ADRs or genitourinary infections, volume depletion, hypotension, and maybe rare but serious events such as ketoacidosis, enabling timely intervention and referral [116,117]. Similarly, CP monitoring programs have demonstrated effectiveness in the early identification of GI related ADRs associated with incretin-based therapies, facilitating prompt communication with prescribers and therapeutic adjustments [118]. Through patient-specific counseling, dose titration support, and preventive guidance, CPs contribute directly to medication safety and real-world pharmacovigilance, reinforcing risk minimization strategies across the continuum of diabetes care (Table 3).

Beyond initial counseling, CPs engage in ongoing monitoring of treatment effectiveness and tolerability, identify adverse outcomes, and support therapeutic adjustments in close collaboration with physicians and other healthcare professionals [24,91,119,120,121]. Qualitative evidence highlights the critical role of CPs in resolving medication-related problems and enhancing medication safety, especially among older adults, despite persistent systemic barriers such as limited access to patient records and time constraints [119]. Expanding CP scope of practice within collaborative care models has been associated with improved clinical outcomes and reduced healthcare burden, reinforcing the value of structured interdisciplinary cooperation with physicians and other healthcare professionals [120]. Collectively, CP involvement in pharmacovigilance activities and interprofessional care pathways strengthens patient safety, promotes rational pharmacotherapy, and supports long-term therapeutic optimization in diabetes management [121].

### 4.5. Supporting Medication Adherence

Pharmacist-led interventions—including medication review, individualized education, behavioral counseling, motivational interviewing, adherence monitoring, and digital follow-up—have demonstrated meaningful improvements in medication adherence and glycemic outcomes among people with T2DM. Evidence from narrative and systematic syntheses indicates that CPs contribute across the diabetes care continuum, from early detection and prevention to therapeutic optimization and long-term monitoring, particularly when interventions are aligned with contemporary clinical guidelines [24]. Community pharmacy-based interventions, most frequently centered on patient education and counseling, have been associated with improved adherence and modest but clinically relevant reductions in HbA1c, with accessibility and continuity of care emerging as key success factors [91]. Data from multidisciplinary and quasi-experimental studies further show that CP involvement enhances health literacy and adherence behaviors, alongside favorable effects on cardiometabolic parameters such as body weight and lipid profiles [115]. Behavioral strategies, including motivational interviewing, have been shown to effectively address adherence barriers and improve patient-reported outcomes, even in settings where glycemic changes are incremental [122]. Stronger glycemic effects were observed in RCTs, such as the MEDIHEALTH program, where structured group-based CP interventions produced sustained improvements in self-efficacy, adherence, and HbA1c over 12 months [123]. Real-world observational evidence further supports these findings, showing significant improvements in adherence rates and multiple glycemic indices, including fasting and postprandial glucose [124]. Overall, the accumulated evidence underscores the growing contribution of CPs to effective, patient-centered diabetes management with both behavioral and clinical impact.

### 4.6. Digital Tools and Technologies for T2DM

Digital pharmaceutical care platforms enable CPs to deliver personalized pharmaceutical care while maintaining longitudinal oversight empowering patients to manage their conditions [138,139]. Furthermore, the recent years, the evolution of digital health tools represent another milestone in successful diabetes treatment providing innovative solutions toward patient empowerment for successful disease management (Table 3). Pharmacist-led digital health interventions—delivered via telephone, web platforms, or mobile applications—appear to offer additional benefits for adherence and glycemic control, particularly when personalized and integrated into routine care [125]. Randomized and cluster trials evaluating digital health-supported lifestyle programs, such as the PRIME program reported by Teoh et al. and Ng et al., further highlight the capacity of pharmacist-supported digital platforms to improve weight outcomes, dietary quality, and cardiometabolic risk factors among individuals with prediabetes [126,127]. As for continuous glucose monitoring (CGM) devices, emerging evidence from pharmacist-led services indicates clinically meaningful improvements in HbA1c, time-in-range, patient empowerment, and service sustainability, while scoping reviews and ongoing prospective studies underscore both the feasibility and the need for stronger real-world evidence [128,129,130,140]. These findings indicate that the introduction of digital health technologies prompts CPs towards a new role as key providers and interpreters of patient-generated health data, with studies evaluating their role to be underway [141].

## 5. Barriers and Perspectives

Despite strong evidence supporting the effectiveness of pharmacist-led interventions in T2DM management, the implementation of structured clinical services in community pharmacies remains limited by several systemic barriers [94] (Table 4). These include inadequate communication and coordination with physicians, staffing shortages, high workload, lack of reimbursement for clinical services, restricted access to patient health records, and insufficient privacy within pharmacy settings. Patient reluctance to engage in pharmacist-led services and persistence under recognition of CPs’ clinical role further impede integration into healthcare systems [119,120,142,143].

From a citizen perspective, socioeconomic factors such as income, insurance status, and the readiness of health systems significantly impact the accessibility of novel therapies. Individuals with lower income or without sufficient insurance are less likely to receive advanced treatments, which can lead to worse health outcomes and perpetuate disparities [144,145,146,147]. To overcome these barriers, policies should lower financial burdens, enhance insurance coverage, and prioritize those most in need. Health systems must deliver person-centered care, invest in education, and regularly update strategies to ensure equitable access to innovative therapies [148].

Additional challenges relate to pharmacists’ professional preparedness. Many CPs report limited training and confidence in managing contemporary antidiabetic therapies, particularly newer drug classes [119,120]. Studies assessing pharmacists’ knowledge of SGLT2 inhibitors have identified only moderate familiarity, raising concerns regarding optimal patient support and safety monitoring [149]. Hence, the need for regularly updated continuing professional development programs focused on novel diabetes therapies and interdisciplinary collaboration is also a necessity [120,150,151,152].

Despite these challenges, accumulating evidence demonstrates that pharmacist-led interventions improve self-management, medication adherence, and optimization of cardiometabolic therapies, while facilitating timely and appropriate adoption of innovative antidiabetic agents. The strongest evidence supports pharmacist involvement in medication reconciliation, adherence support, and early identification and management of ADRs. In contrast, outcomes related to hard clinical endpoints, such as hospitalization and mortality, remain less consistently reported and warrant further investigation through high-quality, adequately powered studies [153,154]. As to socioeconomic factors, CPs can also play a vital role in overcoming socioeconomic obstacles by offering affordable medication options, providing culturally sensitive patient education, and connecting individuals to essential social services, thereby facilitating equitable access to healthcare and medications [148,155,156]. Future efforts should prioritize legislative recognition of expanded pharmacist roles, integration of pharmacists into multidisciplinary care teams, and implementation of reimbursed, standardized diabetes services in community pharmacy settings [24,143,157,158]. Strengthening interprofessional collaboration and providing accredited continuing education in modern and combination diabetes therapies will be essential to fully realize the potential of CPs within contemporary diabetes care and pharmacovigilance frameworks [24,98,125,143,159].

## 6. Materials and Methods

This narrative review was conducted using a structured literature search strategy to identify relevant evidence on (i) contemporary pharmacotherapy for T2DM and (ii) the evolving role of CPs in real-world implementation, medication optimization, and pharmacovigilance. A systematic search was performed in PubMed/MEDLINE, Scopus, Web of Science, and the Cochrane Library, covering publications from January 2015 to December 2025, with the final search conducted in December 2025. Search strategies combined Medical Subject Headings (MeSH) and free-text terms related to T2DM, modern antidiabetic pharmacotherapy, and pharmacist-led interventions, including combinations such as “type 2 diabetes” AND (“GLP-1 receptor agonist” OR “DPP-4 inhibitor” OR “SGLT2 inhibitor” OR “tirzepatide” OR “dual incretin”) AND (“community pharmacist” OR “pharmacist intervention” OR “medication management” OR “pharmacovigilance”). Equivalent adaptations of these search terms were applied across databases.

Eligible studies were required to be published in English, involve adult populations with T2DM, and include international clinical guidelines, RCTs, systematic reviews, meta-analyses, or high-quality observational studies addressing either pharmacotherapeutic outcomes beyond glycemic control or pharmacist-led interventions in diabetes care. Case reports, conference abstracts, editorials without primary data, non–peer-reviewed sources, and studies focusing exclusively on type 1 diabetes or gestational diabetes were excluded. Titles and abstracts were screened, followed by full-text assessment of potentially relevant articles. Screening was conducted by one author (AF), with unclear cases discussed to reach consensus (MS). Evidence was prioritized hierarchically, with international guidelines and large RCTs emphasized for pharmacotherapeutic positioning, systematic reviews and meta-analyses prioritized for pharmacist-led interventions, and observational studies included to contextualize real-world practice. Given the narrative design of the review, no formal risk-of-bias assessment tool was applied, which is acknowledged as a methodological limitation. The narrative design and absence of formal risk-of-bias assessment may introduce selection bias; however, prioritization of guidelines, RCTs and systematic reviews was used to mitigate this limitation.

## 7. Conclusions

The introduction of modern antidiabetic therapies—including GLP-1 receptor agonists, dual GIP/GLP-1 receptor agonists, and SGLT2 inhibitors—has reshaped T2DM management by extending therapeutic benefits beyond glycemic control to cardiovascular and renal protection. However, their rapid and widespread use underscores the need for robust pharmacoepidemiological evaluation and active pharmacovigilance to monitor real-world effectiveness, safety, and long-term outcomes across diverse patient populations. CPs, as highly accessible healthcare professionals, are uniquely positioned to support medication safety through continuous patient engagement, early identification and reporting of ADRs, optimization of drug utilization through medication review, and adherence support within primary care. Integrating pharmacist-led services into formal pharmacovigilance and real-world evidence frameworks can enhance patient safety, improve population-level outcomes, and promote the sustainable implementation of innovative antidiabetic therapies. Targeted education on novel therapies and strengthened interdisciplinary collaboration are essential to advance the role of CPs as integral contributors to safe, effective, and equitable diabetes care.

## Figures and Tables

**Table 1:** Major therapeutic classes for T2DM as to cardiovascular and renal benefit; impact on weight and potential adverse drug reactions. (↑: increase; ↓: decrease; →: leads in).

Drug Class	Molecules	Pharmacological Mechanisms	Administration	Cardiovascular & Renal Protection	Weight Loss	Adverse Effects
DPP-4 Inhibitors	sitagliptin, linagliptin, vildagliptin, saxagliptin, alogliptin	DPP-4 inhibition → endogenous GLP-1/GIP ↑, insulin ↑, glucagon ↓	Oral, once daily; fixed-dose combinations available	HbA1c ↓; CV safety established; linagliptin renal neutral; saxagliptin/alogliptin: HF caution	Neutral	Low hypoglycaemia risk; mild infections; rare pancreatitis, arthralgia
GLP-1 RAs	liraglutide, dulaglutide, semaglutide, lixisenatide, exenatide	GLP-1 receptor activation → ↑ insulin, ↓ glucagon, delayed gastric emptying, appetite suppression	Subcutaneous; daily or weekly	MACE ↓; slowed CKD progression; albuminuria ↓; improved BP, lipids, NAFLD	Moderate to Substantial	GI intolerance; rare pancreatitis; very low hypoglycaemia risk
Dual GIP & GLP-1 RAs	tirzepatide	Dual GLP-1/GIP receptor activation	Subcutaneous; weekly; titration required	Improved cardiometabolic profile; renal benefit signals; BP, lipids ↓	Marked, dose-dependent	GI effects (dose-related)
SGLT2 Inhibitors	dapagliflozin, canagliflozin, empagliflozin	Renal SGLT2 blockade → glucosuria, natriuresis	Oral, once daily	HF hospitalization ↓, CV mortality, CKD progression (with/without T2DM) BP ↓	Mild (2–4 kg)	Genitourinary infections; volume depletion; rare euglycaemic DKA

**Table 2:** PICO-based summaries of systematic reviews and meta-analyses evaluating the impact of community pharmacist interventions on diabetes and metabolic syndrome. (↑: increase; ↓: decrease).

Population (P)	Intervention (I)	Comparator (C)	Key Outcomes (Effect Direction)	Ref.
Adults with diabetes in primary care	Pharmacist-led education and structured medication review	Usual care	HbA1c ↓	[98]
Patients with T1DM and T2DM in community settings	Pharmacist-led pharmaceutical care (medication review, goal setting, physician feedback)	Standard care	HbA1c ↓; care coordination ↑	[99]
Adults with diabetes (global; 59 studies)	Pharmacist-led educational and behavioral interventions	Usual care	Medication adherence ↑; glycemic target attainment ↑	[100]
Community-dwelling adults with diabetes	Pharmacist counseling, structured education, telephone follow-up	Usual care	Medication adherence ↑; HbA1c ↓	[91]
Patients with T2DM	Pharmacist-led education targeting self-care behaviors	Standard care	Self-care behaviors ↑; HbA1c ↓	[101]
Adults with diabetes (36 RCTs; *n* = 5761)	Pharmacist-led care (education, medication review, regular follow-up)	Usual care	HbA1c ↓; adherence ↑; cardiometabolic risk ↓	[102]
Adults with diabetes (ambulatory settings) (24RCTs; *n* = 3610)	Pharmacist-led self-management education and support	Usual care	HbA1c ↓; adherence ↑ Self-management ↑	[103]
Adults with T2DM in Mexico	Pharmacist-led interventions pharmacotherapeutic follow-up patient education, and pharmaceutical care interventions	Usual care	HbA1c ↓; FBG ↓ cardiometabolic risk ↓; Medication adherence; patient education	[104]
Adults with metabolic syndrome (incl. T2DM/prediabetes)	Pharmacist participation in multidisciplinary teams	Non-pharmacist or standard team care	MetS target attainment ↑; reversion to non-MetS ↑; adherence ↑	[105]

**Table 3:** Framework of interventions and impact on care for CPs in the Management of T2DM.

Pharmaceutical Care Domain	Key CP Activities	Impact on Care/Outcomes	References
Patient identification, treatment initiation, and follow-up	Early detection, referral, support of GLP-1 RA/SGLT2i initiation	Improved real-world uptake of evidence-based therapies	[12,24,105,106,107]
MR & optimization	Structured MR; regimen optimization; deprescribing	Reduced DRPs and polypharmacy; improved adherence and medication safety; optimized therapeutic outcomes	[108,109,110,111,112]
Patient education & self-care	Education on medication use, benefits, risks; lifestyle and self-management	Improved adherence; fewer medication errors; improved glycemic control; better quality of life; lower complication rates	[101,108,113,114,115]
Safety monitoring, pharmacovigilance, and ADR management	ADR detection; counseling; dose/titration support; reporting & communication with prescribers	Early safety signal detection; improved tolerability; strengthened real-world pharmacovigilance; coordinated diabetes care	[24,64,65,91,116,117,118,119,120,121,122]
Adherence support	Individualized counseling; motivational interviewing; adherence monitoring; behavioral and digital follow-up	Improved medication adherence; glycemic control; health literacy; lipid profiles	[24,91,100,115,122,123,124]
Digital tools	Digital platforms; reminders; lifestyle apps; CGM services; PV reporting	Improved adherence, HbA1c, weight, dietary quality; enhanced patient empowerment	[125,126,127,128,129,130]

**Table 4:** Key Barriers and Future Directions for Community Pharmacist-Led Interventions in T2DM.

Domain	Key Barriers/Challenges	Impact on Care	Future Directions/Perspectives
Health system integration	Limited communication with physicians; weak integration into PHC	Fragmented care; suboptimal therapy optimization	Formal inclusion of pharmacists in multidisciplinary care teams
Workforce and workload	Staffing shortages; high workload; limited time	Reduced delivery of clinical and safety services	Workforce support; protected time for clinical roles
Reimbursement and policy	No reimbursement for clinical services; limited legislative support	Poor sustainability and scalability of interventions	Reimbursed, standardized diabetes services; regulatory recognition
Infrastructure and data access	Limited access to patient records; insufficient privacy	Incomplete assessment; barriers to pharmacovigilance	Shared electronic records; improved pharmacy infrastructure
Professional preparedness	Limited training in novel antidiabetic therapies	Inconsistent counseling; potential safety gaps	Mandatory, continuous education in modern diabetes therapies
Patient engagement and recognition	Low patient uptake; underrecognition of pharmacist role	Reduced adherence and service utilization	Public awareness; patient-centered engagement strategies
Socioeconomic Factors	Income, insurance status, readiness of health systems	lower income/insurance leads to disparities in access and disease management,	Offer affordable options, culturally sensitive education, connect to social services

## Data Availability

No new data was created.
